# Review of the interaction between lifestyle habits and personality disorders

**DOI:** 10.1192/j.eurpsy.2022.1702

**Published:** 2022-09-01

**Authors:** S. St-Amour, F.-A. Bérubé, L. Cailhol, C. Le Corff

**Affiliations:** 1 Université du Québec à Montréal, Sciences De L’activité Physique, Montréal, Canada; 2 Institut Universitaire en Santé Mentale de Montréal, Clinique Des Troubles Relationnels Et De La Personnalité, Montreal, Canada; 3 Institut Universitaire de Santé Mentale de Montréal, Psychiatry, Montréal, Canada

**Keywords:** Personality disorders, physical activity, Lifestyle, nutrition

## Abstract

**Introduction:**

Individuals with personality disorders have a decreased life expectancy when compared to the general population in particular due to physical illnesses. Many factors can be associated with those physical illnesses such as lack of physical activity and bad nutritional habits. Moreover, physical activity and nutrition (lifestyle) intervention have shown great results in decreasing symptoms and improving condition in affective and anxiety disorders. However, little is known about the relation between lifestyle, and personality disorders.

**Objectives:**

The purpose of this review is to regroup the available information on this topic.

**Methods:**

In February 2021, we searched the literature using 4 databases for articles analyzing the relation between lifestyle and personality disorders. Twenty-one articles were included.

**Results:**

In this review, we found few studies analyzing the relation between lifestyle and personality disorders. Most studies either used lifestyle measures as control variables or did not use such variables at all. Moreover, instruments used to measure lifestyle variables lacked precision at best. Two studies demonstrated a relation between early malnutrition and further development of personality disorders, but those results may be influenced by confounding variables and cannot indicate a clear link between nutrition and personality disorder.

**Conclusions:**

Few evidences are available linking lifestyle to personality disorders in any way. This lack of evidence is surprising considering the multiple benefits individuals with personality disorders could get from it. More studies are needed to thoroughly analyze the impact of lifestyle on personality disorders and vice versa. Those studies need to use validated instruments to provide strong evidence about this relation.

**Disclosure:**

No significant relationships.

